# Management of stable angina pectoris in private healthcare settings in South Africa

**DOI:** 10.5830/CVJA-2018-020

**Published:** 2018

**Authors:** Tlhakudi Pride, John Mathibe Lehlohonolo

**Affiliations:** Division of Pharmacology (Therapeutics), University of KwaZulu-Natal, Durban, South Africa; Division of Pharmacology (Therapeutics), University of KwaZulu-Natal, Durban, South Africa

**Keywords:** revascularisation, angina pectoris, optimal medical therapy (OMT), medical aid scheme, South Africa, private healthcare

## Abstract

**Aim:**

Angina pectoris continues to affect multitudes of people around the world. In this study the management of stable angina pectoris in private healthcare settings in South Africa (SA) was investigated. In particular, we reviewed the frequency of medical versus surgical interventions when used as first-line therapy.

**Methods:**

This was a retrospective inferential study carried out using records of patients in private healthcare settings. All cases that were authorised for reimbursement by medical aid schemes for revascularisation between 2009 and 2014 were retrieved and a database was created. Data were analysed using MicrosoftR Excel and GraphPad PrismR version 5. The differences (where applicable) were considered statistically significant if the p-value was ≤ 0.05.

**Results:**

Nine hundred and twenty-two patients, consisting of 585 males (average age 64.7 years; SD 12.9) and 337 females (average age 65.5 years; SD 14.3), met the inclusion criteria. One hundred and seventy-eighty or 54%, 156 (43%) and 86 (63%) patients with hypertension, hyperlipidaemia and diabetes, respectively, were treated with surgery only. For these patients, percutaneous coronary interventions (PCIs) were significantly (p < 0.0001) preferred first-line interventions over optimal medical therapy (OMT). Four hundred and thirty-six or 47% of all patients studied were managed with surgery only, while only 25% (227) were managed with OMT. It took 60 months (five years) for patients who were treated with OMT before their first surgical intervention(s) to require the second revascularisation. About 71% of patients who received medical therapy were placed on only one drug, the so called sub-optimal medical therapy (SOMT).

**Conclusion:**

The management of stable angina pectoris in private healthcare settings in SA is skewed towards surgical interventions as opposed to OMT. This is contrary to what consistent scientific evidence and international treatment guidelines suggest.

The World Health Organisation (WHO) estimates indicate that in 2010, ischaemic heart diseases were responsible for 7.3 million deaths worldwide, and that 58 million disability-adjusted life years (DALYs) were lost as per the global burden of this disease.^1^ Furthermore, the American Heart Association has reported that about 15.4 million people in the United States of America in 2010 had ischaemic heart diseases.^2^ In South Africa (SA), ischaemic heart disease is one of the 10 leading causes of death.^3^ This is in line with global trends.^4^ However, there is very little epidemiological data about the burden caused by stable angina and the economic implications of the way it is managed in SA (both in the public and private healthcare settings).

Angina pectoris is one of the symptoms of various ischaemic heart diseases that affect the coronary arteries. It is mainly due to atherosclerosis, coronary embolism and/or calcific aortic stenosis.^5^,^6^ Angina is characterised by thoracic pain that occurs as a result of deficiency in blood delivery to the myocardium. Depending on the nature, duration and its responsiveness to medical therapy, angina pectoris may be regarded as stable or unstable.^7^ With the former, the symptoms, which are associated with the extent of physical exertion, are generally responsive to medical therapy. However, in patients with unstable angina, a thoracic pain, which occurs even at rest, is not amenable to medical therapy.^8^

Management of angina pectoris includes non-pharmacological measures, such as lifestyle modifications. For the relief of symptoms, a step-wise management approach or an optimal medical therapy (OMT) is recommended.^9^ For OMT, eligible patients are treated with a triple-drug regimen, which consists of aspirin, beta-blockers, nitrates, calcium channel blockers, potassium channel activators and/or vasodilators, such as nicorandil, sodium channel blockers, such as ranolazine, or 3-hydroxy-3-methylglutaryl-CoA (HMG-CoA) reductase inhibitors, such as simvastatin.^10^-^12^

Revascularisation and other surgical procedures play a life-saving role for the majority of patients with angina.^13^-^15^ Commonly used surgical techniques include percutaneous coronary interventions (PCIs), bare-metal stents (BMS), coronary artery bypass grafting (CABG) and drug-eluting stents (DES). Myocardial infarction causes death in many untreated and asymptomatic angina patients.^5^

Treatment of unstable angina, an emergency condition, is undisputed.^16^ However, management of stable angina remains the elephant in the room.^17^ In several developed countries, about 85% of revascularisations were performed on stable coronary patients who could have been well controlled on OMT.^18^ This continues to happen despite overwhelming evidence from studies such as the Clinical Outcomes Utilizing Revascularisation and Aggressive drug Evaluation Trial (COURAGE trial) pointing to the contrary.^19^ Unfortunately, in developing countries such as SA, there is insufficient evidence on how stable angina is managed, especially in private healthcare settings.

The main aim of this study was to investigate management patterns of stable angina pectoris in private healthcare settings in SA. In particular, we sought to: investigate how frequent medical versus surgical interventions were used as first-line therapy in patients with stable angina pectoris; assess the rationale of choice of surgical interventions over medical interventions; and assess the need for subsequent surgical interventions, if surgical therapy was preferred over medical interventions.

## Methods

This was a retrospective inferential study conducted using a database of reimbursement by a medical scheme in the private healthcare sector. An inferential data analysis aims to deduce whether the observed pattern(s) will hold in another population(s) as opposed to individuals.^20^ All patients diagnosed with ischaemic heart disease (IHD) or angina and authorised for reimbursement between 2009 and 2014 were included for analysis.

To determine the duration between the first and second interventions, the month and year in which the second intervention was done was subtracted from the month and year in which the first intervention was carried out. In those instances where the month was not indicated or only the year was indicated, it was assumed that the intervention was done in January.

The protocol was given full ethics approval by the Research Ethics Committee, University of KwaZulu-Natal (Ref BE 398/14).

## Statistical analysis

Variables were characterised using 95% confidence intervals (CIs). Means with standard deviations for continuous variables were used to analyse proportions/ratios for the categorical data. Binary logistic regression was used to identify independent associations between the first intervention (revascularisation) versus the second intervention, and between OMT versus revascularisation. Associations were considered statistically significant if p ≤ 0.05. The GraphPad Prism version 5.0 with the freeware package R version 2.13.1 was used for statistical manipulations and analyses. The outliers were included, unless otherwise stated.

## Results

A total of 922 patient files were included in the analysis in this study. There were 585 (63%) males and 337 (37%) females, with average ages of 64.7 (SD ± 12.9) and 64.7 (SD ± 14.3) years, respectively. Angina-related co-morbidities included hypertension, hyperlipidaemia and diabetes, present in 45, 36 and 20% of patients, respectively. These co-morbidities, when they existed separately, were spread evenly between males and females. However, as shown in Table 1, co-existing incidences of hypertension and hyperlipidaemias were significantly (p < 0.05) twice as high in males as females. The incidence of other conditions in males compared to females was not statistically significantly different.

**Table 1:** The relationship between various co-morbidities and gender in patients with stable angina pectoris

*Co-morbidities*	*Males n (%)*	*Females n (%)*	*Both n (%)*
Hypertension (H)	74 (8.03)	52 (5.64)	126 (13.67)
Hyperlipidaemia (HL)	44 (4.77)	21 (2.28)	65 (7.05)
Diabetes (D)	48 (5.21)	24 (2.6)	72 (7.81)
H + HL + D	30 (3.25)	13 (1.41)	43 (4.66)
H + HL	136 (14.75)	66 (7.16)	202 (21.91)
H + D	31 (3.36)	16 (1.74)	47 (5.1)
HL + D	17 (1.84)	5 (0.54)	22 (2.39)
Other*	205 (22.23)	140 (15.18)	345 (37.42)
Total	585 (63.44)	337 (36.55)	922 (100)

*Other co-morbidities were asthma, chronic obstructive pulmonary disease, hypotension and heart failure.

One hundred and seventy-eighty or 54%, 156 (43%) and 86 (63%) patients with hypertension, hyperlipidaemia and diabetes, respectively, were treated with surgery only. For these patients, PCIs were significantly (p < 0.0001) the preferred first-line interventions over OMT. A combination of OMT and surgery as a preferred intervention accounted for only 8% of all patients studied. As a result, a total of 436 (47%) of all patients studied were managed with surgery only, while only 25% (227) were managed with OMT, as shown in Table 2. About 71% of patients who received medical therapy were placed on only one drug, the so-called sub-optimal medical therapy (SOMT).

**Table 2:** Use of OMT versus surgical interventions in patients with stable angina pectoris with different co-morbidities

*Co-morbidities*	*OMT n (%)*	*OMT plus PCIs n (%)*	*PCIs only n (%)*
Hypertension (H)	16 (1.74)	13 (1.41)	52 (5.64)
Hyperlipidaemia (HL)	90 (0.98)	4 (0.43)	41 (4.45)
Diabetes (D)	7 (0.76)	5 (0.54)	33 (3.58)
H + HL + D	7 (0.76)	6 (0.65)	22 (2.39)
H + HL	68 (7.38)	23 (2.49)	83 (9)
H + D	9 (0.98)	7 (0.76)	21 (2.28)
HL + D	5 (0.54)	3 (0.33)	10 (1.08)
Other*	25 (2.71)	15 (1.63)	174 (18.87)
Total**	227 (24.6)	76 (8.2)	436 (47.3)

*Other co-morbidities were asthma, chronic obstructive pulmonary disease, hypotension and heart failure.

**20% (183) of patients, although diagnosed with stable angina pectoris, did not receive any treatment.

In some cases, reasons or motivation for not using OMT as the first-line intervention were provided. For example OMT was considered inappropriate/contra-indicated in 3.5, 5.2, 5.2, 3.8, 1.5 and 0.8% of patients with asthma, chronic obstructive pulmonary disease, hypotension, heart failure, poor lung function and uncontrolled diabetes, respectively. The use of a beta-blocker was stopped in 1.2% of patients due to intolerance, asthma, wheezing, poor lung function and depression. Largevessel occlusion, heart failure, peripheral vessel disease and single-vessel disease were stated as motivating factors for revascularisation. Unfortunately and without explanation, 20% (183) of patients, although diagnosed with stable angina pectoris, did not receive any treatment.

Fifty-six per cent (or 520 of all patients studied, that is 333 males and 187 females) were treated with one type or another of revascularisation with or without medicine. Subsequently, 139 (42%) males and 94 (50%) females who were treated with revascularisation needed a second surgical intervention. However, the differences in the need for the second surgical intervention between males and females were marginally significantly different (p = 0.06). Thereafter, about 18% (25) of males and 21% (20) of females who received the second surgical invention needed a third revascularisation to be carried out. A total of 16 patients (eight males and eight females) needed more than three surgical interventions.

**Fig. 1:**
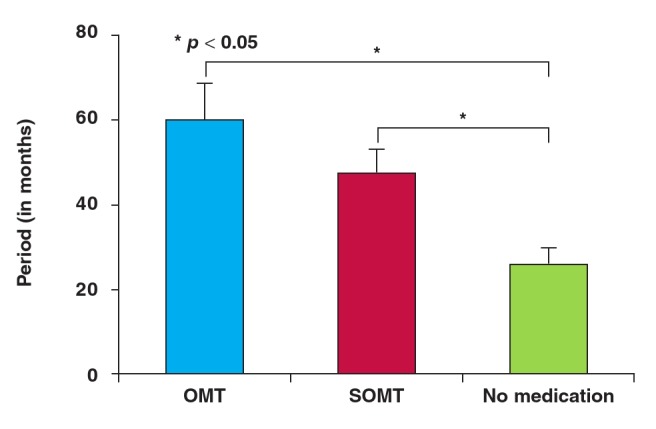
Period (in months) it took for groups of patients with stable angina to require second surgical intervention. OMT: optimal medical therapy; SOMT: sub-optimal medical therapy.

As depicted in Fig. 1, it took 60 months (five years) for patients who were treated with OMT before their first surgical intervention to require the second revascularisation. Those who received SOMT and those who did not receive medication at all before their first surgery took 48 and 26 months, respectively, to require the second revascularisation. The differences (i.e. 34 months) between those who were on OMT and those who did not receive any medical therapy before their first surgical intervention were statistically significant (p < 0.001).

Similarly, the differences (22 months) between those who were on SOMT and those who did not receive any medical therapy before their first surgical intervention were statistically significant (p < 0.05). The specific type of the first surgical intervention had a significant (p < 0.05) impact on how long it took for the second revascularisation to be needed. For example, it took 138 months (about 11.5 years), 46 months (nearly four years) and 18 months for patients who received CABG, BMS and DES, respectively, to require the second revascularisation.

## Discussion

The main findings of this study indicate that OMT is pivotal in the management of stable angina pectoris. This is in support of several recent studies that have shown that there were no differences between PCIs and OMT with regard to the all major outcomes in patients with stable angina pectoris.^21^ What is more exciting and novel about our findings, in addition to corroborating other recent findings such as those reported by Iqbal et al., is that OMT reduces the need for subsequent PCIs when used before or together with an appropriate surgical intervention(s).^22^ More importantly, this study has shown that OMT lengthens the period between surgical interventions. However, the average age (65 years) of patients in this study might have played a role in these findings. Recently, Won and colleagues reported that PCIs were more beneficial than OMT in patients with stable angina pectoris, aged 75 to 85 years old.^23^

It was regrettable, as shown by the findings of our study, that 75% of patient aged 65 years old (on average), who might have benefited immensely, were not treated with OMT as the initial management approach. Furthermore, the use of OMT in this study was significantly less than the 44% reported in the COURAGE study.^19^ However it was much better than the 17% reported from the New York State Registry.^21^ Therefore, it means that the vast majority of medical practitioners in private healthcare settings in South Africa still prefer surgical interventions as the initial management approach for stable angina pectoris, although there is strong evidence to the contrary.

The barriers to effective implementation of clinical guidelines and their uptake into routine clinical practice are well documented worldwide.^24^,^25^ For example, Grol and Grimshaw reported that absence of facilities, lack of feasibility, old routines, heavy work-load, as well as no immediate risk of consequences for non-compliance were the main barriers for poor implementation of evidence.^26^

The latter offers a possible explanation for the lack of implementation of the findings of the COURAGE trial^19^ in private healthcare settings in South Africa, as reported in this study. In these settings, there are generally no immediate consequences for medical practitioners not adhering to clinical guidelines. This happens because other than the strict requirements set by medical aid schemes in South Africa, mostly each medical practitioner relies on his/her own expert judgement. More importantly, ‘professional pride and payer profit’ have a big impact on ‘perspectives on optimal care and the best method for improving health care’.^27^

Therefore, it is also possible that OMT was less favoured in private healthcare settings because of its minimal financial benefits for medical practitioners, compared to surgery. As a result, the majority of cardiologists in private healthcare settings, as was recently reported by Mohee and Wheatcroft, continue to underestimate the benefits of OMT in patients with stable angina pectoris.^28^

There are some limitations to this study. As it often the case with other retrospective studies, there were missing data from the files of patients studied. Most notably, we could not assess the impact OMT on survival because of missing mortality data. However, there is a low prevalence of mortality due to stable angina pectoris.^29^,^30^ Therefore it is unlikely that lack of data on survival rates in the population studied had a significant impact on the findings of this study.

## Conclusion

Compelling evidence suggests that OMT should be the initial management approach in patients with stable angina. Therefore a reasonable approach is to optimise OMT and reserve coronary revascularisation for mainly older patients who are sub-optimally controlled on medical therapy, or for patients who are at high risk of major adverse cardiac events.
